# Y-chromosome target enrichment reveals rapid expansion of haplogroup R1b-DF27 in Iberia during the Bronze Age transition

**DOI:** 10.1038/s41598-022-25200-7

**Published:** 2022-12-01

**Authors:** Carla García-Fernández, Esther Lizano, Marco Telford, Íñigo Olalde, Rafael de Cid, Maarten H. D. Larmuseau, Marian M. de Pancorbo, Francesc Calafell

**Affiliations:** 1 Department of Medicine and Life Sciences, Institute of Evolutionary Biology (UPF-CSIC), Universitat Pompeu Fabra, Dr. Aiguader 88, 08003 Barcelona, Spain; 2 Institut Català de Paleontologia Miquel Crusafont, Universitat Autònoma de Barcelona, Cerdanyola del Vallès, Spain; 3 BIOMICs Research Group, University of the Basque Country UPV/EHU, Vitoria-Gasteiz, Spain; 4 Ikerbasque—Basque Foundation of Science, Bilbao, Spain; 5 Genomes for Life-GCAT Lab, Germans Trias i Pujol Research Institute (IGTP), Badalona, Spain; 6 Laboratory of Human Genetic Genealogy, Department of Human Genetics, KU Leuven, Leuven, Belgium; 7 ARCHES–Antwerp Cultural Heritage Sciences, Faculty of Design Sciences, University of Antwerp, Antwerp, Belgium; 8 Histories Vzw, Gent, Belgium

**Keywords:** Population genetics, Phylogenomics, Biological anthropology, Population genetics

## Abstract

The Y chromosome can yield a unique perspective into the study of human demographic history. However, due to the repetitive nature of part of its sequence, only a small set of regions are suitable for variant calling and discovery from short-read sequencing data. These regions combined represent 8.9 Mbp or 0.14% of a diploid human genome. Consequently, investing in whole-genome sequencing to resolve Y-chromosome questions is poorly efficient. Here we use, as an alternative, target enrichment technology to greatly increase sequencing effectiveness, validating and applying the technique to 181 males, for 162 of whom we obtained a positive result. Additionally, 75 samples sequenced for the whole genome were also included, for a total sample size of 237. These samples were chosen for their Y chromosome haplogroup: R1b-DF27. In the context of European populations, and particularly in Iberia, this haplogroup stands out for its high frequency and its demographic history. Current evidence indicates that the diffusion of this haplogroup is related to the population movements that mark the cultural Bronze Age transition, making it remarkably interesting for population geneticists. The results of this study show the effects of the rapid radiation of the haplogroup in Spain, as even with the higher discriminating power of whole sequences, most haplotypes still fall within the R1b-DF27* paragroup rather than in the main derived branches. However, we were able to refine the ISOGG 2019–2020 phylogeny, and its two main subbranches, namely L176.2 and Z272, which present geographical differentiation between the Atlantic and Mediterranean coasts of Iberia.

## Introduction

The non-recombining portions of the genome, namely mitochondrial DNA (mtDNA) and the non-recombining portion of the Y chromosome (NRY) are especially informative. Although each behave as a single locus, they have been used in multiple applications, such as forensic genetics^1^, genetic genealogy^2^ and population genetics^3^. Precisely because of their lack of recombination, maximum parsimony phylogenies can be constructed in mtDNA and the NRY with relative ease, and, combined with the observation of the geographical provenance of each haplotype, these phylogenies can be turned into phylogeographical frameworks that can be used to trace sex-specific admixture patterns among present or past human populations^4^.

In the particular case of the Y chromosome, although it contains tens of thousands of SNPs and small indels that have been the basis for reconstructing its phylogeny^5^ (see also the citizen-curated phylogeny at https://isogg.org/tree/index.html), they are embedded in a highly repetitive genome structure that makes working with the entire Y chromosome particularly difficult. This led to the definition of the so-called callable region^6^, actually a set of regions in the Y chromosome that could be sequenced and variants called in it without interference from the numerous repetitive structures, and that was also Y-specific and not shared with the X chromosome. The length of the callable region is ~ 8.9 Mb, that is, about 0.3% of the haploid size of the entire genome. Traditionally, uniparental studies based on massively parallel sequencing data were a secondary result from whole-genome sequencing efforts, meaning that, if the focus was on the Y chromosome, 99.7% of the sequencing effort was wasted^7^. However, the development of target enrichment assays that increase the proportion of a specific genomic region before sequencing allows designing projects to specifically study the region of interest.

Previous studies have highlighted the potential of applying target enrichment techniques to obtain Y-chromosome sequences in different scenarios: in cases in which the objective is to increment the number of modern Y-chromosome samples in population genetics studies^8^, or where Y-chromosome information is difficult to retrieve because of molecular damage, as in ancient DNA^9,10^. We have designed probes to capture the 8.9 Mb of the Y-chromosome callable region or target region (TR). These 8.9 Mb are not continuous along the chromosome but are separated in nine different regions, mostly present in the X-degenerate and the ampliconic fractions of the Y chromosome. We have then applied this method to study the R1b-DF27 branch of the Y-chromosome phylogeny and its involvement in the Bronze Age population movements in Western Europe and particularly in the Iberian Peninsula.

The Bronze Age transition in the 3rd millennium BC was a key moment of social and demographic transformations in Europe. The Bell-beaker complex was one of the major cultures that marked this transition, and was predominant in the Western and South-Western parts of the continent. Its spread was a complex process involving cultural diffusion and demographic migrations with a variable balance. Individuals associated with the Bell-Beaker complex were genetically heterogeneous, with a cline of ancestries related to the Eastern Steppe, European Middle Neolithic, and Copper Age groups^11^. Other cultural groups were also present in the Early Bronze Age, such as the El Argar culture of SE Spain^12^. In contrast, their Y-chromosome pool was more homogeneous and had a high predominance of a single haplogroup, R1b-M269^11^, associated with the arrival of Steppe-related ancestry to central Europe by 3000BC. Remarkably, R1b-M269 is still the most frequent Y-chromosome haplogroup in Western Europe^13^, showing that the expansion of the Bell-Beaker complex had an important role in its dissemination. This expansion left strong footprints on the genetic and cultural landscapes of the Iberian Peninsula, and during the Bronze Age, 40% of the Iberian genetic ancestry was related to central European Bell-Beaker complex-associated groups^14^. This impact was even more pronounced in the Y chromosome, due to an almost complete genetic replacement of the diverse Copper Age lineages with R1b-M269^14^. One branch of this haplogroup, R1b-P312, is the most abundant in West Europe^13^ and in turn, it splits into three main subbranches: U152, frequent in Northern Italy and the Alps regions^15^; L21, more restricted to Ireland and the British Islands^16^; and DF27, predominant in the Iberian Peninsula^17–20^. In this project, we focus on R1b-DF27, which has a high frequency in the Peninsula, reaching 40% of the Y-chromosome haplogroups both in Spain and Portugal, while it is much rarer elsewhere^21^. It has been hypothesized that the origin of this lineage lies in the Northern part of Spain around 4000 ya^17^, and it seems to have diverged shortly after into sublineages with potential geographic differentiation. Specifically, R1b-L176.2 appears more frequently in the East, and R1b-Z220 tends to peak in the North-Central part of the Peninsula.

In the present project, we analyze the demographic history of the R1b-DF27 lineage in West Europe, and more specifically in Spain, to refine its internal structure and phylogeography. We have combined modern individual sequences, either from preexisting whole genomes, or from captured Y-chromosome sequences. Whole genomes were obtained within the GCAT project, which aims to describe genomic and phenotypic variation in residents in Catalonia^22^. We also aimed to demonstrate that by designing and implementing an in-house protocol to capture 8.9 Mb of the Y-chromosome we could optimize a technique to our specific sample type and interests and obtain a good-quality cost-effective dataset on R1b-DF27 Y-chromosome sequences.

## Results and discussion

### Validating the performance of target enrichment

**Figure 1:**
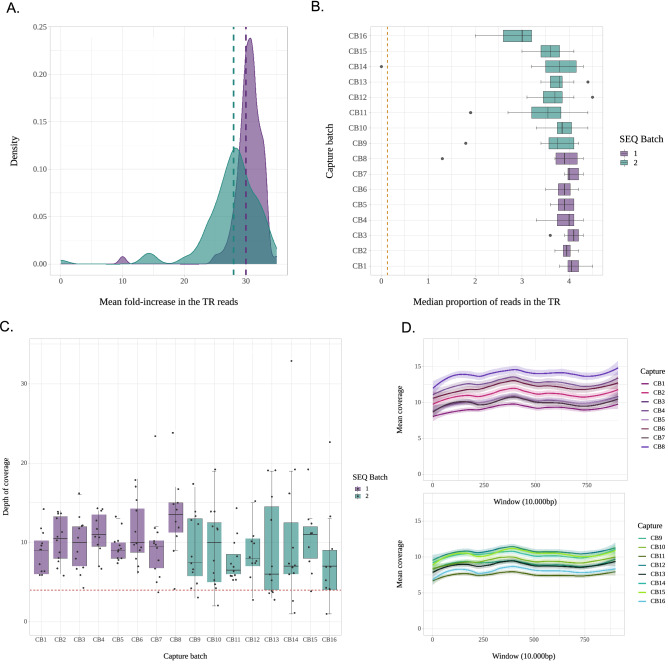
Parameters of capture performance. (**A**) Distribution of the mean fold- increase in the proportion of reliable reads in the Target Region per sequencing batch. The vertical lines correspond to mean values. (**B**) The median proportion of reliable reads in the Target Region per capture batch. The brown line corresponds to this proportion in one non-enriched sample. (**C**) Median depth of coverage in the Target Region for each capture batch and 95% confidence intervals. (**D**). Distribution of mean coverage across 902 genomic windows in the target region by sequencing batch.

We generated DNA libraries for 181 individuals and captured the target region (TR) of the Y chromosome for each individual. As a result, we obtained a total of 35 million reads, divided into 17 million for the first batch (SEQ1) and 18 million for the second batch (SEQ2) (Table S1). Out of the total reads mapped against the GRCh37 reference of the human genome, 11% corresponded to duplicates generated during the amplification steps of the library preparation and capture protocol. This does not imply a meaningful increase from the usual value in WGS without enrichment (9.8% in the GCAT dataset we used to supplement our sequences, see below). To evaluate the performance of the capture experiments, we calculated the fold-increase in the proportion of reads covering the TR (Fig. 1A). We observed that this value was 29 times higher in our captured sequences, and that the median proportion of reads covering the TR was 4% in our dataset (Fig. 1B), compared to 0.13% in non-enriched GCAT samples. All capture batches presented similar proportions of reads in the TR. We observed that one library in capture batch 14 (LB11) was not enriched in the TR and was eliminated from the final dataset.

The median depth of coverage in our target region was 9.5X (± 4.9) and showed little variance across capture batches. As an additional quality control, all samples with a median coverage < 4X were filtered out (Fig. 1C). Thus, we discarded three samples from SEQ1 and 14 samples from SEQ2. The coverage distribution across the captured regions appears homogeneous through all of its length, which supports the robustness of the design of the baits (Fig. 1D). The enrichment factor (EF) can be used as a way to evaluate the performance of the capture experiment, as it takes into account the size of target spaces and the total genome size. In our study the mean EF was 24, in line with other capture studies targeting chimpanzee DNA from faecal samples^23^, or regions of interest in dusk-biting mosquito genomes^24^.

In summary, we obtained 162 Y-chromosome TR sequences at a median 9.5X coverage for a sequencing effort that would have produced just one 1X Y chromosome in a classical WGS setting.

### New insights into the phylogenetic structure of R1b-DF27

Our total sample size was 237 individuals, after the addition of 75 GCAT sequences^22^ (see the “Methods” section) to the 162 samples that we enriched for the Y-chromosome callable region. We found a total of 22 different haplogroups in our dataset. From the total of 237 individuals, 30 were ancestral for DF27 and belonged instead to P312* (3), U152 (9), L21(9), DF19 (7), one to Y17209, and one was the R1a individual we used as an outgroup. Therefore, DF27 itself was found in 207 individuals. With this final dataset, we proceeded to the analysis of the phylogenetic structure and geographic distribution of R1b-DF27. We investigated the phylogenetic structure of these sequences by constructing a BEAST tree (Supplementary Fig. 1), identifying haplogroups as defined in the ISOGG 2019–2020 R1b tree with Y-Lineage Tracker^25^, and manually inspecting the sequences. It is quite apparent that the R1b-DF27 haplogroup does not seem to present a solid internal structure, which complicates the inference of a robust tree phylogeny reflecting the known SNP phylogeny. Forty different branches stem directly from the R1b-DF27 root; 19 of those represent singleton sequences, and only seven correspond to basal mutations already described in the ISOGG 2019–2020 R1b tree and detected with Y-Lineage Tracker. Thus, the R1b-DF27* paragroup (as for the SNPs present in the ISOGG 2019–2020 v 15.23 R1b tree) was carried by 73 individuals, or slightly over one third of the sample. This first result is probably a consequence of the rapid expansion of DF27 in the Iberian Peninsula, as was hypothesized in Solé-Morata et al.^17^; indeed, this seems to be a general trend for the whole of R1b-M269 in W Europe^26^.

However, we were able to detect SNPs that had not been typed in previous works^17–20^ (such as A432 and CTS9952), and we could refine the phylogeny given by ISOGG in 2019–2020. Indeed, the branch marked as R1b1a1b1a1a2a7 ~ (with the tilde indicating a set of mutations with an uncertain position) was previously designated as a single branch stemming from DF27 and defined by a set of nine mutations (CTS6519.1/S4247.1, CTS11567/Z2572, DF79, DF81, DF83, DF84, S453/Z224, Z222, S360/Z223). Instead, we found the derived states for only four of those SNPs (namely, CTS6519.1/S4247.1, CTS11567/Z2572, DF81 and Z222). Individuals carrying the derived state at any of these SNPs were ancestral for all of the SNPs branching directly from R1b-DF27 (including all others in R1b1a1b1a1a2a7 ~), and thus constitute four new basal branches within R1b-DF27. However, these four SNPs do lie in separate branches in the privately managed Y-full tree (https://www.yfull.com/tree).

**Figure 2:**
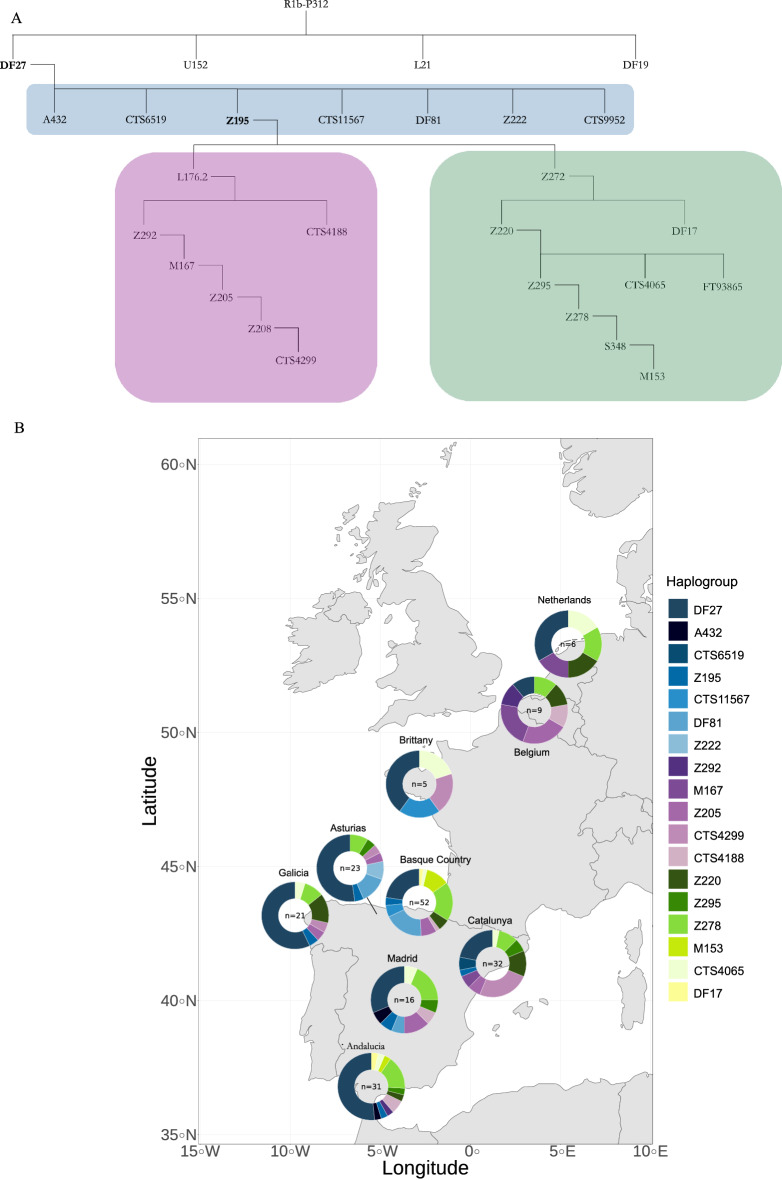
On top, schematic phylogeny of the SNPs in the ISOGG 2019–2020 tree found in our samples. Note that, as discussed in the text, our results can be used to refine the ISOGG 2019–2020 phylogeny. Bottom, haplogroup frequencies in our samples, as defined by the terminal SNP (i.e., all other branch-defining SNPs below that SNP and mapping to the TR were found to be ancestral). Map source: mapplots 1.5.1 R package.

Haplogroup R1b-DF27 is divided into two main branches, characterized by Z220 and L176.2 respectively, both of which split from R1b-Z195, which derives directly from R1b-DF27. According to ISOGG 2019–2020, R1b-Z220 is actually a branch of Z272. Moreover, our sample contained individuals carrying Y chromosomes that were derived for DF17, a subbranch that stems directly from Z272 and is ancestral for Z220. In the branch defined by L176.2 (R1b1a1b1a1a2a1b), a set of mutations is also annotated by ISOGG 2019–2020 as of uncertain position (R1b1a1b1a1a2a1b1a1a ~ containing Z205, S362/Z208, CTS8289 and CTS4299). We could unequivocally place them under M167/SRY2627, and a hierarchical structure emerged within them, with a number of individuals carrying the derived allele for Z205 but being ancestral for the remaining alleles, and others being derived for Z205, S362/Z208, CTS8289, but not CTS4299, and finally, others carrying the derived allele in all four SNPs. Again, this is also presented in the Yfull tree. A schematic phylogeny based on the previously described SNPs and our findings is shown in Fig. 2A.

Haplogroup divergence time estimates (Table 1) were ~ 1–1.5 Kya older both than those expected based on the fossil record^11^ and than those based on STR diversity^17^. Ancient DNA inference dates are a minimum threshold for the actual emergence of the mutations, but the difference we observed is most likely the result of discrepancies in the mutation rates used. We can observe, however, that the rapid expansion of DF27 lineages was followed by rapid diversification of the L176.2 and Z272 branches, that present older ages than sister branches of Z195 like CTS11567, CTS6519.1 or DF81.

**Table 1:** Branch divergence times inferred with BEAST for DF27 and its sublineages, and 95% highest posterior density intervals. NA: branches that are not recovered as monophyletic in the BEAST tree (Supplementary Fig. 1). Rho TMRCA estimates (± standard deviation) and confidence intervals (computed from the 95% confidence interval for mutation rates).

Branch	Divergence time (ya BP), BEAST	95% HPD	Mutation TMRCA (rho)	95% CI (mutation rate)
DF27	6566	5997–7403	5173 ± 72	(4571, 5868)
A432	NA	NA	3992 ± 62	(3527, 4480)
CTS11657	2564	1359–4260	4028 ± 63	(3560, 4570)
CTS6519	3512	1979–5887	5100 ± 70	(4507, 5785)
DF81	4141	2948–5717	4368 ± 65	(3876, 4975)
Z222	4043	2509–6021	3548 ± 60	(3136, 4025)
Z195	5176	4279–5983	5374 ± 73	(4749, 6096)
L176.2	6296	6214–6393	4505 ± 67	(3981, 5110)
Z292	NA	NA	4173 ± 65	(3688, 4733)
M167	5190	4698–5720	4027 ± 63	(3559, 4568)
Z205	4290	3630–5045	3941 ± 63	(3488, 4470)
Z208	4491	3817–5369	3366 ± 58	(2975, 3818)
CTS4299	4304	3569–4969	3414 ± 58	(3017, 3872)
CTS4188	5127	3627–6606	4553 ± 67	(4024, 5165)
Z272	5987	5549–6460	4832 ± 70	(4271, 5482)
Z220	NA	NA	4710 ± 69	(4162, 5343)
Z295	5037	4649–5423	4043 ± 64	(3573, 4587)
Z278	4188	3831–4540	3941 ± 63	(3483, 4471)
S348	3742	3241–4512	3440 ± 59	(3040, 3902)
M153	3567	2881–3758	2920 ± 54	(2580, 3312)
CTS4065	4760	3696–5699	3918 ± 63	(3462, 4444)
DF17	4821	3389–6071	3647 ± 60	(3223, 4137)

We also used the rho statistic^27^ to estimate the age of the main branches of the R1b-DF27 phylogeny (Table 1). Although ages estimated by both methods are highly correlated (Pearson’s r = 0.866), rho estimates are on average 16% lower, bringing them closer to those reported by Solé-Morata et al.^17^ based on Y-STRs, and to the likely appearance of R1b-DF27 in the ancient DNA record^3^. The discrepancy may result from the different goals of each method: while BEAST was devised to infer branch lengths and divergence times, rho estimates TMRCAs, which are, per se, slightly younger, since they refer not to the origin of a branch, but to the time since variation started accruing in that branch.

Next, we estimated the age of R1b-DF27 from the variation found in several geographical regions in Spain and North-West Europe (Table 2). The estimated age was highest in the Basque Country, Catalonia and NW Europe, and lowest in Central Spain. However, differences are small and cannot be used to pinpoint a place of origin for R1b-DF27, since it is likely that the initial Bronze Age event carried R1b-DF27 throughout Iberia within a short timeframe.

**Table 2:** Rho TMRCA estimates (in years ago before the present) for R1b-DF27 in different geographical regions. See Table S2 for the definition of each region.

Region	TMRCA	95% CI (mutation rate)
Andalusia	4922 ± 70	(4349 ± 66, 5583 ± 75)
Asturias	5052 ± 71	(4465 ± 67, 5730 ± 76)
Catalonia	5424 ± 74	(4793 ± 69, 6152 ± 78)
Central Spain	4673 ± 68	(4130 ± 64, 5301 ± 73)
Basque Country	5473 ± 74	(4804 ± 69, 6167 ± 79)
Galicia	4991 ± 71	(4411 ± 66, 5662 ± 75)
NW Europe	5307 ± 73	(4690 ± 68, 6020 ± 76)
Pooled	5173 ± 72	(4571 ± 68, 5868 ± 77)

### R1b-DF27 in the ancient DNA record

We have reanalyzed the ancient DNA Y chromosome sequences reported as R1b-DF27 by visually inspecting their pileup files and comparing them with the SNPs in our phylogenetic reanalysis (see Table S3). The reconstruction of the prehistorical trajectory of R1b-DF27 is complicated by the fact that DF27 itself was not among the 1240 K SNPs included in the in solution capture protocol that has been used to produce most of the recent boom in aDNA genomic results^28^. Thus, the presence of R1b-DF27 is inferred by the observation of its derived branches, such as Z195. That is the case of the oldest observations of R1b-DF27, in the Early Bronze Age (EBA) of Sicily, in which a genotype for DF27 could not be produced, but the derived allele of the parental Z195 SNP is present in two samples dated between 2399 and 2153 calBCE^29^. Recently, though, it has been discovered that in the Argaric site of La Almoloya (SE Spain) all the male individuals for which a genotype could be produced were indeed derived for Z195^12^, the oldest of which dated at 2000–1750 calBCE. While no other Early Bronze Age examples of R1b-Z195 have been found in Sicily or the rest of Italy, they abound in Spain at: Can Roquetes (Catalonia)^14^, Llanos de Betxí (València)^14^, Puntal de los Carniceros (València)^12^. R1b-DF27 is rare in Sicily today: although direct estimates of its frequency do not seem to have been published, its frequency is theoretically capped by the frequency of R1b-P312(xL21, U152) at 4.25%^30^. Still, whether these haplogroups originated in Iberia and were brought to S Italy via a network of maritime trade, or they travelled in the opposite direction, is yet open to speculation^12^.

The oldest direct observation of R1b-DF27 originates from the EBA site of Diamond Cottage, in SW England, dated at 2200–1400 calBCE^31^. However, this individual is not directly dated, and the wide range is derived from the archaeological context. Thus, it might be the case that Cueva de los Lagos (La Rioja, N Spain)^32^, dated at 1600–1300 BCE is older. Other Iberian EBA DF27 examples are Valdescusa (La Rioja)^14^, La Requejada (Valladolid)^14^, and Naveta des Tudons (Menorca)^29^. Currently, the only prehistoric observation of one of the main R1b-Z195 branches is R1b-L176.2 in La Almoloya, where one individual carried the derived state of Z198 (which is in the same branch as L176.2). Curiously, both R1b-L176.2 and R1b-Z272 have been found in Vikings. The only instance of R1b-Z272 and its derivatives in ancient DNA comes from a Viking site in Denmark^33^. Vikings in England, Sweden and Denmark were derived for the other main branch, R1b-L176.2, two of them being R1b-Z205 (and thus, also derived for R1b-M167)^33^. In modern Scandinavian populations, R1b-DF27 might be rare, although precise estimates do not seem to have been produced: R1b-M167 is absent in Denmark, Götland and N Sweden^34^, while an upper limit for the R1b-DF27 frequency, namely R1b-P312(xU152,L21) yields 5.4% in Denmark and 2.2% in S Sweden^13^.

### The two main branches within DF27 have different geographic distributions

We analyzed the frequency of the R1b-DF27 branches in the different geographic areas of Spain and North-West Europe to refine their phylogeographic distribution. The most frequent subgroup is the paragroup R1b-DF27*, which is highly prevalent in the western part of Spain: Galicia (57%), Andalucía (52%), Asturias (43%), and Central Spain (39%) (Fig. 2B, Table S4). The lowest frequencies of this paragroup are found in the Basque Country (23%), Catalonia (22%) and Belgium (11%). Note that these percentages, as all of those given in this section, are over the number of DF27 chromosomes. Since the frequency of those varies, particularly between Spanish and non-Spanish samples, the proportions over the total number of Y chromosomes are bound to be much lower in the latter. In any case, the high frequency of R1b-DF27* Y chromosomes agrees with a rapid expansion scenario of the lineage.

Other than the two main branches (see below), we found six other previously named branches stemming directly from DF27. The most notable is DF81 (7% overall), which reached a frequency of 19% of the DF27 population from the Basque Country. This subhaplogroup is the most frequent in the area and is also present in Asturias (17%) and Madrid (6%) but it is absent elsewhere. The five other branches are rarer, with a combined frequency of 5% and each restricted to one or at most two populations.

Only five individuals (2%) carried Z195 and were not derived either for Z272 or L176.2. The Z272 branch contains, as mentioned above, both the Z220 and DF17 branches; it should be considered that the latter was not genotyped by Solé-Morata et al.^17^, and such haplogroups would appear as Z195* in that paper. Z272 is slightly more frequent in the Basque Country (42%) than elsewhere (Galicia, 34%, Catalonia and Andalucía, 31%). It is also present in Belgium and the Netherlands. Within this branch, one subbranch shows a particular geographic distribution: M153, which was already described by Underhill et al. in 2000^35^ and that was assumed to be restricted to Basques, is indeed found in 6/53 of our Basque sample, with just two other examples elsewhere.

The L176.2 branch is abundant in Catalonia (37%), while its frequency does not exceed 12% elsewhere in Spain. Instead, it is more frequent in our non-Spanish samples: Belgium (55%), Brittany (20%), and the Netherlands (17%). Within L176.2, SRY2627 (M167) had also been discovered two decades ago, by Hurles et al.^36^ in both Basques and Catalans. Our results as well as those produced by Solé-Morata et al.^17^ point to Catalonia as the region with the highest frequency (37% in our case, but also four out of nine Belgian samples). However, we could point out that ten of the 12 Catalan SRY2627/M167 individuals carry Y chromosomes that are derived also for other SNPs, particularly the terminal CTS4299 (Fig. 2A,B).

### Genetic diversity within R1b-DF27 is geographically homogeneous

We found 4,509 SNPs in the 207 Y chromosomes in our sample that belonged to the R1b-DF27 haplogroup; all of these individuals carried different haplotypes and were therefore unique. Within geographical regions, measures of diversity such as nucleotide diversity and the mean number of pairwise differences were small (consistent with the relatively young age of this haplogroup) and similar to each other, although Central Spain showed a slightly reduced diversity, while Catalonia and NW Europe were slightly more diverse (Table 3). This pattern echoes the trend observed for R1b-DF27 age estimates, which, as noted above, were younger in Central Spain and older in Catalonia and NW Europe. As a measure of the skew of the site frequency spectrum towards rarer alleles (which results in the star-like tree R1b-DF27 presents), we have computed Tajima’s D, which shows very negative values (< − 2.4 in all regions).

**Table 3:** Measures of diversity in R1b-DF27 chromosome sequences. S: number of SNPs; ${\uppi }$ : nucleotide diversity (× 1000); MPD: mean nucleotide pairwise differences; D, Tajima’s D (all tests with *p* < 0.0001). (*): the total sample size also includes four individuals from two populations (three individuals from Aragón and one from Murcia) that were not pooled into larger regions and were not analyzed separately given their small sample sizes.

Region	N	S	$\pi$	MPD	D
Andalusia	31	868	0.0071 ± 0.0035	62.78 ± 27.84	− 2.766
Asturias	23	670	0.0073 ± 0.0037	65.06 ± 29.13	− 2.616
Catalonia	32	860	0.0077 ± 0.0038	68.37 ± 30.26	− 2.632
Central Spain	23	614	0.0067 ± 0.0034	60.02 ± 26.9	− 2.606
Basque Country	53	963	0.0073 ± 0.0036	65.09 ± 28.52	− 2.517
Galicia	21	576	0.0072 ± 0.0036	64.1 ± 28.81	− 2.484
NW Europe	20	582	0.0075 ± 0.0038	67.13 ± 30.22	− 2.471
Pooled	207(*)	4509	0.0072 ± 0.0035	65.74 ± 28.46	− 2.947

**Figure 3:**
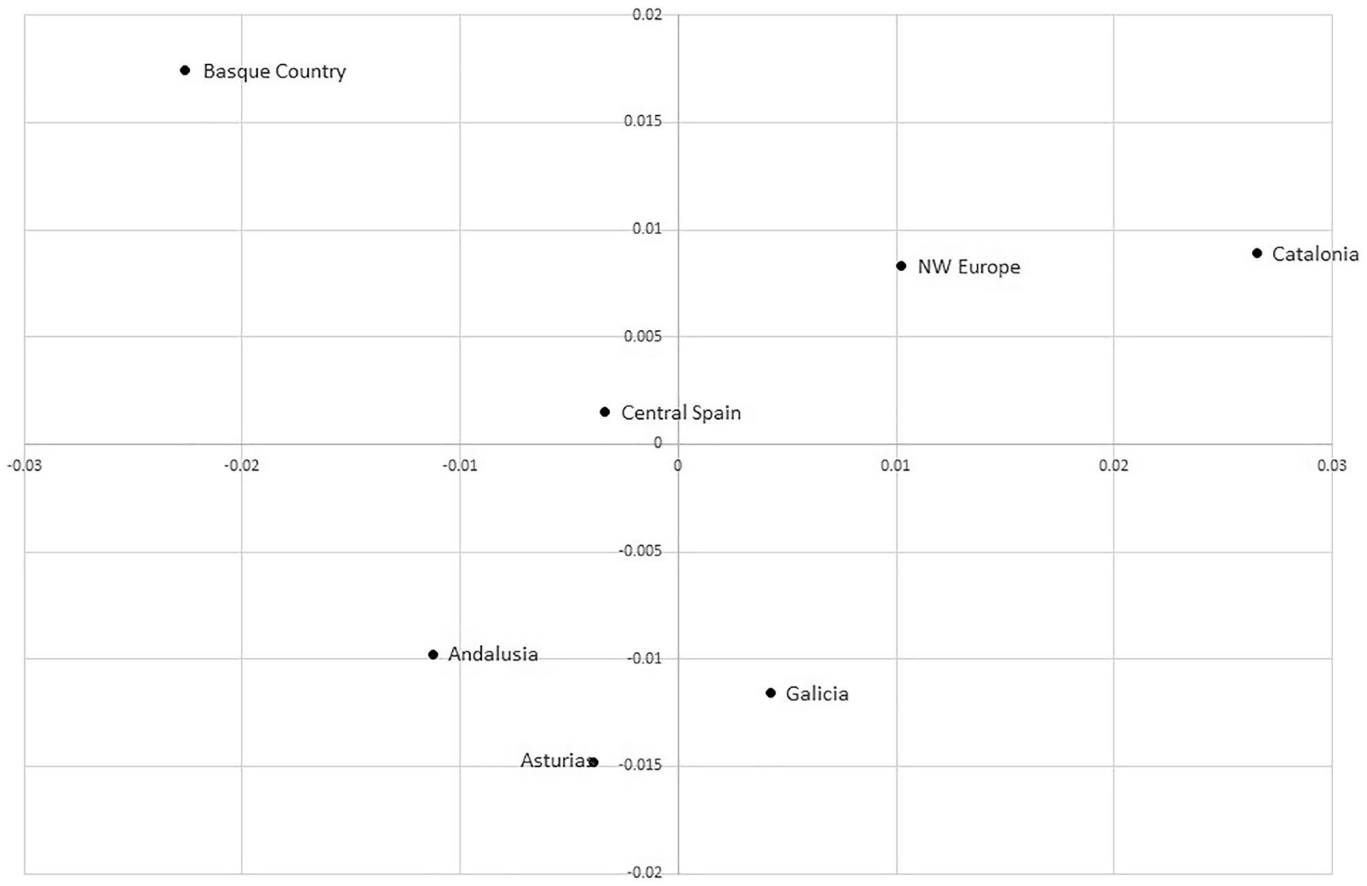
MDS plot of the φst distance among geographical regions. Stress was 1.1%.

Differentiation among regions was measured with AMOVA; 1.51% of the total variation was found among regions (*p* < 0.0001). Although this proportion is statistically significantly different from zero, it is difficult to assess its relevance, since we could not find in the literature comparable estimates for inter-regional, within-haplogroup, sequence-based *F*_*ST*_ values. Pairwise distances (φ_ST_) were computed among regions (Table 4) and plotted with MDS (Fig. 3). The largest distance in the matrix, and in the MDS plot, was found between the Basques and Catalans, with NW Europeans leaning into the latter. This is quite likely the reflection of the relative frequencies of the two main branches of R1b-DF27, namely R1b-Z272, which is more abundant in Basques, and R1b-L176.2, more prevalent in Catalonia and NW Europe.

**Table 4:** Pairwise φ_ST_ distance among geographical regions.

	Andalusia	Asturias	NW Europe	Catalonia	Central Spain	Basque Country	Galicia
Andalusia	0	0.00805	0.00927	0.0244	− 0.00339	0.01645	0.0006
Asturias	0.00805	0	0.01231	0.02435	0.00435	0.02055	0.00577
NW Europe	0.00927	0.01231	0	0.00215	0.00093	0.02039	0.00326
Catalonia	0.0244	0.02435	0.00215	0	0.01356	0.03741	0.01291
Central Spain	− 0.00339	0.00435	0.00093	0.01356	0	0.00842	− 0.00103
Basque Country	0.01645	0.02055	0.02039	0.03741	0.00842	0	0.02161
Galicia	0.0006	0.00577	0.00326	0.01291	− 0.00103	0.02161	0

### Revisiting the origin of R1b-DF27

Taking into account both the frequency of R1b-DF27, which is found in > 40% of Iberian males but declines abruptly to < 10% in western Europe north of the Pyrenees, and is rare elsewhere^18–20^ , and that STR variation linked to R1b-DF27 was greater within Iberia^17^, it was postulated that NE Iberia is the most likely place of origin of DF27^17^. The present results show indeed that nucleotide diversity is marginally higher in NE Iberia than elsewhere, and that whole branches of the R1b-DF27 phylogeny, particularly R1b-Z272, are restricted to Iberia. However, ancient DNA, and the fact that nucleotide diversity is not significantly lower in NW Europe compared to Iberia, do not rule out the possibility that R1b-DF27 originated elsewhere in Western Europe, but expanded and radiated in the north of the Iberian Peninsula, where it replaced the local paternal lineages to a great extent. Both ancient and extant DNA point to the Bronze Age expansions as the cause for the spread of R1b-DF27 throughout Western Europe and particularly into Iberia.

A main caveat in our study design is sampling: sample sizes were extremely low for some Iberian populations, and key areas such as Portugal, and especially France, could not be sampled. Still, in both cases R1b-DF27 frequencies are known and are compatible with our interpretation of the current results. Portugal showed subhaplogroup frequencies similar to those in northern and western Spain^17^, while France echoes the patterns in W Europe^17^. A more granular sampling of France, particularly in the southwest, would be required to fill the gap in our sampling and to increase the precision and certainty about the birthplace of R1b-DF27.

## Materials and methods

### Sampling and endogenous content estimation

Samples were collected from previously typed individuals belonging to R1b-DF27 haplogroup, or R1b-P312 when downstream SNP genotypes were not available. Individuals were selected to cover the peninsular area of Spain and to represent the periphery of the distribution of this haplogroup, in Brittany, Belgium and the Netherlands. All subjects were volunteers who had signed an informed consent form; this research was carried out in accordance with the principles stated in the Declaration of Helsinki. This project was reviewed and approved by the Parc de Salut Mar Comitè d’Ètica en Investigació Clínica IRB, with reference 2019/8900/I, on January 15th, 2020. GCAT sequences were used after the approval by the Hospital Germans Trias i Pujol IRB, ref. PI-19–081, on April 5th, 2019. For all samples, DNA had previously been extracted from saliva. The relative amount of Y chromosome per sample was assessed by measuring the number of SRY copies with qPCR assays using SYBR™ Green Master Mix following the conditions in Table 5. The same male DNA sample extracted from blood was used as a reference standard for the entire project. Five sequential dilutions of this sample were used to calculate a basal standard curve. Each DNA sample, including the standards, was tested in triplicate.

**Table 5:** SRY primers and Master Mix for the qPCR.

SRY primers (5′—> 3′)	
Forward	CATGAACGCATTCATCGTGTGGTC
Reverse	CTGCGGGAAGCAAACTGCAATTCTT

Master Mix	1X
Supermix 5X	4 µl
Primer F	0.4 µl
Primer R	0.4 µl
ddH2O	14.2 µl
DNA	1 µl

Additionally, 75 whole genome sequences of men carrying R1b-DF27 were retrieved from the GCAT project^22,37^; sequences are available from the European Genome-Phenome Archive (EGA) at https://ega-archive.org/datasets/EGAD00001008201. Volunteers in this project were chosen from among public health service users residing in Catalonia.

### Library preparation and target enrichment

Libraries were constructed with double-inline barcodes following the BEST protocol^38^ with the minor modifications introduced by Fontserè et al.^23^. The outcome of the library preparation was quantified with Qubit™ and Agilent 2100 Bioanalyzer. 181 libraries with a DNA concentration above 15 ng/µl and a fragment size distribution between 150 and 400 bp were selected for the subsequent experiments. These libraries were distributed in 16 capture pools according to their relative content of Y-chromosome copies. RNA probes for capture were designed with Agilent SureSelect Custom Target Enrichment Baits, to cover the 8.9 Mb of the target region. Captures were performed following Agilent protocol^39^ with minor modifications as described in Fontserè et al.^23^. One round of hybridization was done in each of the pools. Final captured pools were quantified with Qubit and Bioanalyzer and sequenced in four lanes of HiSeq X with 150 bp paired-end kits in Macrogen (Seoul, South Korea).

### Data processing

Samples were demultiplexed with Sabre into the 181 libraries, and the adapters were removed with Trimmomatic 0.35^40^. They were then mapped with BWA 0.7.15^41^ against the GRCh37 version of the human reference genome. Base quality scores were recalibrated with GATK 3.7^42^ and PCR-duplicates removed with Picard tools 2.8.3. At this point, the target region was selected, and its coverage per sample was calculated with GATK Depth of coverage tool^42^. Variants were called following GATK best practices recommendations^43^, using Haplotype Caller in the haploid mode and Genotype GVCFs. During variant calling, samples from the GCAT^22^ project belonging to DF27 haplogroup were incorporated into the dataset. The final capture and WGS datasets were filtered by quality scores (mapping quality, variant quality and strand bias) coverage^44^, and missingness (SNP missing < 5%).

Our final dataset comprised 237 individuals and 6348 Y- chromosome SNPs (Table S5).

### Capture performance

The proportion of reads in the target region was calculated as the number of reliable reads (i.e. passing quality filters) on target by the total number of reliable reads. This proportion was used to estimate the mean fold-increase compared to the same proportion in whole-genome sequencing samples. The Enrichment Factor was calculated as described in Hernández-Rodríguez et al^45^:

$$EF=\frac{\frac{Reliable \,on-target \,reads}{Total \,reads \,sequenced}}{\frac{Target \,region \,size (8.9 Mb)}{Genome \,size (6 Gb)}}$$

The homogeneity of the coverage of the target region was assessed by dividing the region into 902 genomic windows of 10 Kbp each, estimating the median coverage per window and sample, and finally averaging the coverage for each capture and window.

### Phylogenetic analyses

By comparing the number of derived alleles in capture vs. whole-genome sequences, evidence of reference bias in the former was observed in ten sequence fragments, which were removed from further analysis. These comprised 28,301 bp in total and contained 292 SNPs. Only one of these SNPs was in the ISOGG 2019–2020 v. 15.23 tree, namely CTS12440, which, in the ISOGG tree, defines a clade in haplogroup E rather than R1b. Thus, the removal of these regions is unlikely to have affected the phylogenetic power of our study.

A maximum-likelihood phylogenetic tree was inferred using RAxML 8.2.4^46^ software, and an R1a individual was used as a root. The substitution model used for the analysis was GTRGAMMA with a random seed. The resultant tree was visualized and rooted with FigTree1.4.3^47^. The assignment of haplogroups was made with and yHaplo™^48^ and Y-Lineage Tracker^25^, with versions of ISOGG 2016 and 2019 respectively. Y-Lineage Tracker was also used to calculate haplogroup frequencies in the dataset.

The BEAST 1.7 software^49^ was used to perform Bayesian inference on the coalescent dates. The evolutionary model selected was HKY and the mutation rate of 0.76 × 10^−9^^50^, 95% confidence intervals of the divergence times were estimated using the uncertainty of the mutation rate (0.67–0.86 × 10^−9^). The used clock rate was constant and as a tree prior a random starting tree was selected. The analysis was run for 15 million iterations, with a burn-in of 1,500,000 and a logging frequency of the chains of 1500. To avoid investing large amounts of time and computational resources, invariant sites were estimated and added to the XML file as in Hallast et al.^51^. Five independent runs of BEAST were combined using Logcombiner and annotated using Treeannotator. The final tree was visualized with FigTree^47^.

The rho statistic was measured using the same mutation rate as in BEAST, 0.76 × 10^−9^ mutations/site/year, which, over 8.9 Mb of the sequence produced, translates to 147.84 years/mutation. To compute rho, we counted for each haplotype the number of nucleotide differences from the median haplotype. Note that, as per Saillard et al.^27^, the standard deviation of rho reduces to $\sqrt {\uprho }$ when, as is the case in our sample, the absolute frequency of each haplotype is 1. The 95% CI of the mutation rate estimate, that is, 0.67–0.86 × 10^−9^ mutations/site/year, or 130.65–167.7 years/mutation, was used to provide a 95% CI for the age estimate.

Measures of molecular diversity were estimated with Arlequin 3.5. The same software was used to perform the AMOVA analysis, and to compute Tajima’D, with a *p*-value estimated from 100,000 neutral simulations.

## Supplementary Information

Supplementary Information 1.

Supplementary Information 2.

Supplementary Information 3.

Supplementary Information 4.

Supplementary Information 5.

Supplementary Information 6.

Supplementary Information 7.

Supplementary Information 8.

## Data Availability

The Y chromosome sequences used in this paper are deposited at EGA (https://ega-archive.org/datasets/EGAD00001008202).
